# High-pressure synthesis, crystal structure, and magnetic properties of KSbO_3_-type 5*d* oxides K_0.84_OsO_3_ and Bi_2.93_Os_3_O_11_

**DOI:** 10.1088/1468-6996/15/6/064901

**Published:** 2014-12-29

**Authors:** Yahua Yuan, Hai L Feng, Youguo Shi, Yoshihiro Tsujimoto, Alexei A Belik, Yoshitaka Matsushita, Masao Arai, Jianfeng He, Masahiko Tanaka, Kazunari Yamaura

**Affiliations:** 1 Superconducting Properties Unit, National Institute for Materials Science, 1-1 Namiki, Tsukuba, Ibaraki 305-0044, Japan; 2 Graduate School of Chemical Sciences and Engineering, Hokkaido University, North 10 West 8, Kita-ku, Sapporo, Hokkaido 060-0810, Japan; 3 Beijing National Laboratory for Condensed Matter Physics and Institute of Physics, Chinese Academy of Sciences, Beijing 100190, People’s Republic of China; 4 Materials Processing Unit, National Institute for Materials Science, 1-2-1 Sengen, Tsukuba, Ibaraki 305-0047, Japan; 5 International Center for Materials Nanoarchitectonics (WPI-MANA), National Institute for Materials Science, 1-1 Namiki, Tsukuba, Ibaraki 305-0044, Japan; 6 Materials Analysis Station, National Institute for Materials Science, 1-2-1 Sengen, Tsukuba, Ibaraki 305-0047, Japan; 7 Computational Materials Science Center, National Institute for Materials Science, 1-1 Namiki, Tsukuba, Ibaraki 305-0044, Japan; 8 Synchrotron X-ray Station at SPring-8, National Institute for Materials Science, Kohto 1-1-1, Sayo-cho, Hyogo 679-5148, Japan

**Keywords:** high-pressure synthesis, osmium oxide, KSbO_3_-type, KOsO_3_, Bi_3_Os_3_O_11_

## Abstract

5*d* Solid-state oxides K_0.84_OsO_3_ (Os^5.16+^; 5*d* ^2.84^) and Bi_2.93_Os_3_O_11_ (Os^4.40+^; 5*d* ^3.60^) were synthesized under high-pressure and high-temperature conditions (6 GPa and 1500–1700 °C). Their crystal structures were determined by synchrotron x-ray diffraction and their 5*d* electronic properties and tunnel-like structure motifs were investigated. A KSbO_3_-type structure with a space group of *Im*-3 and *Pn*-3 was determined for K_0.84_OsO_3_ and Bi_2.93_Os_3_O_11_, respectively. The magnetic and electronic transport properties of the polycrystalline compounds were compared with those obtained theoretically. It was revealed that the 5*d* tunnel-like structures are paramagnetic with metallic charge conduction at temperatures above 2 K. This was similar to what was observed for structurally relevant 5*d* oxides, including Bi_3_Re_3_O_11_ (Re^4.33+^; 5*d* ^2.66^) and Ba_2_Ir_3_O_9_ (Ir^4.66+^; 5*d* ^4.33^). The absence of long-range magnetic order seems to be common among 5*d* KSbO_3_-like oxides, regardless of the number of 5*d* electrons (between 2.6 and 4.3 per 5*d* atom).

## 1. Introduction

Crystalline KSbO_3_-type [1] or comparable-type solid-state oxides are attractive for their possible applications in ionically conductive and electrocatalytic materials [2–5]. This prospect may be related to the presence of tunnel motifs in the crystal structure of these oxides [6]. Furthermore, structurally related La_4_Ru_6_O_19_ has received considerable attention because of its role in novel electronic transport in non-Fermi-liquids [7, 8]. The KSbO_3_-type family of solid-state oxides is currently an emergent subject in the field of inorganic chemistry. They can be used to develop advanced technologies for energy-related applications and to help understand correlated electron properties.

The KSbO_3_-type family of oxides consists of several compositional variants have been synthesized and characterized by the similar tunnel motifs, such as the following:

• *AM*O_3_: KSbO_3_ [1], KIrO_3_ [9], BaOsO_3_ [10], KBiO_3_ [4, 11], AgBiO_3_ [12];
• *A*_3_*M*_3_O_11_: La_3_Ir_3_O_11_ [13], Bi_3_Ru_3_O_11_ [3], La_3_Ru_3_O_11_ [7, 14], Bi_3_Os_3_O_11_ [15, 16], Bi_3_Mn_1.9_Te_1.1_O_11_ [17], Bi_3_Re_3_O_11_ [18], Bi_3_CrSb_2_O_11_ [19], Bi_2_La*M*Sb_2_O_11_ (*M* = Cr, Mn, Fe) [19], NaBi_2_Sb_3_O_11_ [20];
• *A*_2_*M*_3_O_9_: Sr_2_Re_3_O_9_ [18];
• *A*_4_*M*_6_O_19_: La_4_Re_6_O_19_ [18], Pb_6_Re_6_O_19_ [18], Ba_4_Os_6_O_18_Cl [21], La_4_Os_6_O_19_ [21], Sr_4_Ru_6_ClO_18_ [22], and La_4_Ru_6_O_19_ [7, 8].

Most of these examples were synthesized at ambient pressure, whilst high-pressure heating has led to successful syntheses of additional compounds, which include NaOsO_3_ [15], Bi_3_Mn_3_O_11_ [17, 23, 24], Bi_3_Ge_3_O_10.5_ [25], Ba_2_Ir_3_O_9_ [26], and Bi_3_Cr_2.91_O_11_ [27].

Our recent studies have focused on the synthesis of solid-state osmium oxides in order to develop 5*d* electronic properties and 5*d* materials for possible advancements in the field of spintronics and related scientific devices [28–31]. During our attempted syntheses of compositionally new osmium oxides under high-pressure and high-temperature conditions, an additional oxide K_0.84_OsO_3_ was synthesized (at 6 GPa). The polycrystalline compounds K_0.84_OsO_3_ was studied by using synchrotron x-ray diffraction and magnetic and charge transport measurements. The refined crystal structure indicated that the crystalline oxide has a KSbO_3_-type structure and shares a tunnel structural motif with a related Os oxide Bi_2.93_Os_3_O_11_ [15, 16]. Herein, we report the synthesis, crystal structure, and primary electrical and magnetic properties of the newly synthesized KSbO_3_-type oxide K_0.84_OsO_3_ and compare those with the properties of the structurally comparable oxide Bi_2.93_Os_3_O_11_.

## 2. Materials and methods

Polycrystalline K_0.84_OsO_3_ was synthesized by a solid-state reaction method in a belt-type high-pressure and high-temperature apparatus (Kobe Steel, Japan), in which a pyrophyllite cell was used to produce a quasi-hydrostatic environment at an elevated pressure [32]. The starting materials Os (99.95%, Heraeus Materials Technology) and KO_2_ (O_2_-45.6%, yellow powder, Sigma-Aldrich) were mixed at a molar ratio of 1:2 in a glove box under argon. The mixture was sealed in a platinum capsule, followed by heating in a compressed pyrophyllite cell at 1500 °C for 1 h. The capsule pressure was maintained at 6 GPa during the heating process. The capsule was then quenched to ambient temperature within a minute by cutting off the electric power supply before releasing the pressure. The final product was a dense pellet of part of it was ground in an agate mortar and pestle. The powder was then rinsed in an ultrasonic water bath multiple times to remove any residue. The high-pressure method is helpful in reducing the risk of human exposure to possible presence of toxic OsO_4_ during the synthesis.

Polycrystalline Bi_2.93_Os_3_O_11_ was similarly prepared using fine powders of Bi_2_O_3_ (99.999%, Kojundo Chem. Lab, Japan) and OsO_2_ (Os-83%, Alfa Aesar) in the high-pressure apparatus. A small amount of an oxygen source (KClO_4_, 99.5%, Kishida Chem) was added to a stoichiometric mixture of the starting materials. The elevated pressure was maintained at 6 GPa during the heating process at 1700 °C for 1 h. Residues in the final product (including KCl) were removed in a water bath.

The final products were characterized by synchrotron x-ray diffraction (SXRD) using a large Debye–Scherrer camera at the beam line BL15XU in the SPring-8 synchrotron radiation facility, Japan [33]. The diffraction profiles were collected at room temperature between 2 *θ* of 3° and 81° at 0.003° intervals using a monochromatized beam (*λ* = 0.65 298 Å or 0.40 025 Å). The wavelength was confirmed by measurements of a standard material (CeO_2_). Each powder was placed into a Lindenmann glass capillary (inner diameter: 0.1 mm) and rotated during the measurements. The SXRD profiles were analyzed by a Rietveld method using the program RIETAN-FP [34].

The dc magnetic susceptibility (*χ*) of the compound was measured in the Magnetic Property Measurement System (MPMS, Quantum Design) between 2 and 395 K in an applied magnetic field of 10 kOe. Each powdered compound was loosely gathered in a sample holder and cooled to the temperature limit. The magnetic field was then applied to the holder. The holder was gradually warmed to 395 K (zero-field cooling, ZFC), followed by cooling in the field (field cooling, FC). The isothermal magnetization of the compounds was also measured in the apparatus with a magnetic field range between –70 and 70 kOe at 5 K. The specific heat *C*_p_ of a piece of the physically compressed bulk material was measured in the Physical Property Measurement System (PPMS, Quantum Design) between 2 and 300 K. In the apparatus, the electrical resistivity (*ρ*) of a pellet piece was measured by a 4-terminal method using platinum wires and a silver paste.

The K content of polycrystalline K_0.84_OsO_3_ was determined by inductively coupled plasma spectrometry. Water-rinsed fine powder was used in the analysis and the average K content was found to be 0.837(7) in accordance with the formula unit.

First-principles calculations of the electronic state of the stoichiometric hosts KOsO_3_ and Bi_3_Os_3_O_11_ were performed by a generalized gradient approximation [35] of the density functional theory. The WIEN2k program [36] was used, which was based on the full-potential augmented plane-wave method. The muffin–tin radii were chosen to be 2.4 atomic unit (au) for K, 2.2 au for Bi, 1.9 au for Os, and 1.6 au for O atoms. The spin–orbit interaction was included as a perturbation to the scalar-relativistic equations. The cut-off wave vector *K* was fixed at *RK* = 8, where *R* is the smallest muffin–tin radius (i.e. 1.6 au). The Brillouin zone integration was approximated by the tetrahedron method with 294 *k* points in an irreducible zone for KOsO_3_ and 76 *k* points for Bi_3_Os_3_O_11_. We assumed that K atoms occupy the K1 site for KOsO_3_ and Bi atoms occupy Bi1 (8*e*) and Bi2 (4*b*) sites for Bi_3_Os_3_O_11_ to avoid fractional occupation.

## 3. Results and discussion

**Figure 1.**
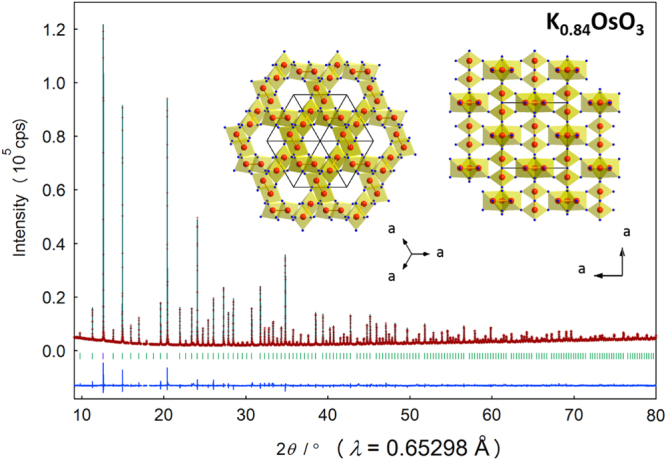
Rietveld analysis of the SXRD pattern for K_0.84_OsO_3_ at room temperature. Markers and solid lines show the observed and calculated profiles, respectively, and the difference is shown at the bottom of the figure. The expected Bragg reflections are marked by the small bars; and the reflections analyzed under partial profile relaxation are marked in purple color [34]. The proposed crystal structure is presented in the inset, in which Os and O atoms are drawn as large red and small blue balls, respectively. K atoms are not shown for clarity.

The crystal structure of K_0.84_OsO_3_ was characterized well by a cubic model with a space group of *Im*-3, similar to KBiO_3_ and AgBiO_3_ [4, 11, 12]. Figure 1 shows the SXRD pattern for K_0.84_OsO_3_ at room temperature. Rietveld analysis conducted on the pattern with the cubic model resulted in a well-refined profile with *R* indices below 7%. The refined cubic lattice parameter was *a* = 9.47 164(1) Å, which is smaller than the corresponding cubic parameters of KBiO_3_ (10.0194(6) Å) and AgBiO_3_ (9.7852(2) Å). The smaller ionic radius (0.575 Å) of Os(V) in an octahedral environment than that of Bi(V) (0.76 Å) may account for this observation [37]. We concluded that a reasonable fit was established; the final structural parameters, including the refined atomic coordinates, are listed in table 1. Although the true chemical composition was slightly under-stoichiometric (K_0.84_OsO_3_), we analyzed the pattern without considering the small amount of K deficiencies that we were unable to refine. However, the thermal parameters for all atoms remained within a reasonable level regardless of the K deficiencies. The small amount of deficiencies may have been distributed almost equally over the three crystallographic K sites, minimizing impact on the analysis.

**Table 1.** Structural parameters of K_0.84_OsO_3_.

Atom	Site	*g*	*x*	*y*	*z*	*B* (Å^2^)
K1	16*f*	0.5	0.3429(2)	=*x*	=*x*	1.65(9)
K2	16*f*	0.213	0.2818(5)	=*x*	=*x*	1.4(2)
K3	2*a*	0.296	0	0	0	1.4(4)
Os	12*e*	1	0.5	0.14 047(3)	0	0.237(4)
O1	12*d*	1	0.3482(7)	0	0	1.6(1)
O2	24 *g*	1	0.3597(5)	0.2935(4)	0	0.74(7)

Note. The space group is *Im*-3 (no. 204), *a* = 9.47164(1) Å, *Z* = 12, *V* = 849.718(2) Å^3^, and *d*_cal_ = 6.50 g cm^−3^. *R* Indices were *R*_wp_ = 3.55%, *R*_p_ = 2.47%, *R*_B_ = 6.58%, and *R*_F_ = 5.57%. The bond distances of Os–O in the OsO_6_ octahedra were *d*(Os-O1) = 1.972(6) Å (×2), *d*(Os-O2) = 1.954(6) Å (×2), and *d*(Os-O2) = 1.966(6) Å (×2). BVS(Os) = +4.63, In which BVS = 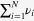, ν_*i*_ = exp[(*R*_0_−*d*_*i*_)/*B*], *N* is the coordination number, *B* = 0.37 and *R*_0_(Os^5+^) = 1.868 [38] (BVS, bond valence sum).

The refined crystal structure is illustrated in the inset of figure 1. The structural view indicates that an Os atom occupies the center of the octahedron. The view clearly shows a characteristic tunnel motif as was observed for other KSbO_3_-type and related oxides. In the octahedra, each edge or corner is shared by neighboring octahedra, causing the shortest Os–Os distance to be 2.6610(4) Å. The distance is 8.7% longer than the bonded Ru–Ru distance of La_4_Ru_6_O_19_ (2.448 Å) and 12.5% shorter than the non-bonded Ru–Ru distance of La_3_Ru_3_O_11_ [7, 8]. The distances may suggest that Os–Os bonding has formed. It can be noted that the ionic size of Os is slightly larger than that of Ru [37]; however, the ionic size difference is unlikely to affect the observation.

**Figure 2.**
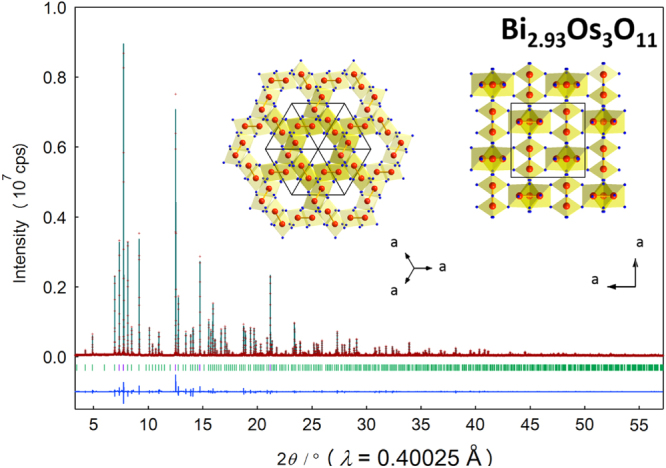
Rietveld analysis of the SXRD pattern for Bi_2.93_Os_3_O_11_ at room temperature. Markers and solid lines show the observed and calculated profiles, respectively, and the difference is shown at the bottom. The expected Bragg reflections are marked by the small bars; and the reflections analyzed under partial profile relaxation are marked in purple color [34]. The proposed crystal structure is presented in the inset, in which Os and O atoms are drawn as large red and small blue balls, respectively. K atoms are not shown for clarity.

Figure 2 shows the SXRD pattern for Bi_2.93_Os_3_O_11_ measured at room temperature (as well as the refined pattern). In the refinement, Bi_2.93_Os_3_O_11_ was assumed to be isostructural to Bi_3_Mn_3_O_11_ [24]; fractional atomic coordinates for Bi_3_Mn_3_O_11_ were tested in early refinements. The Bi1 atom was found to be disordered as in Bi_3_Mn_3_O_11_ [24] and Bi_3_GaSb_2_O_11_ [39]. Eventually, a refinement with an occupation factor (*g*) of 1/3 for Bi1 and 1 for Bi2 resulted in a negative thermal parameter (*B*) for O1; however, the *B*(O1) was positive when we allowed the refinement of *g*(Bi1) and *g*(Bi2). A small amount of vacancies was therefore suggested at these sites; the refined composition was Bi_2.93_Os_3_O_11_. The under-stoichiometric composition may be connected to the presence of a small amount of impurities in the compound. We also note that splitting of Bi2 from the ideal 4*b* site (0,0,0) to 8*e* site (*x*, *x*, *x*) resulted in slightly better *R* factors at *x* = 0.0029(4). Note that the shortest Os–Os distance is 2.5653(3) Å, comparable with the Os–Os distance in K_0.84_OsO_3_. Details of the refinement are summarized in table 2.

**Table 2.** Structural parameters of Bi_2.93_Os_3_O_11_.

Atom	Site	*g*	*x*	*y*	*z*	*B* (Å^2^)
Bi1	24 *h*	0.3255(6)	0.3890(7)	0.3844(9)	0.3736(4)	0.39(2)
Bi2	8*e*	0.4887(9)	0.0029(4)	=*x*	=*x*	0.531(18)
Os	12 *g*	1	0.38 677(5)	0.75	0.25	0.161(4)
O1	8*e*	1	0.1501(5)	=*x*	=*x*	0.04(14)
O2	12 *f*	1	0.5823(9)	0.25	0.25	0.36(14)
O3	24 *h*	1	0.6018(6)	0.2426(5)	0.5343(6)	0.13(7)

Note. The space group is *Pn*-3 (no. 201) at origin choice 2, *Z* = 4, *a* = 9.35993(2) Å, and *V* = 820.007(4) Å^3^. *R* Indices were *R*_wp_ = 6.42%, *R*_p_ = 4.03%, *R*_B_ = 3.52%, and *R*_F_ = 1.75%. The bond distances of Os–O in the OsO_6_ octahedra were *d*(Os-O3) = 1.991(4) Å (×2), *d*(Os-O2) = 1.991(5) Å (×2), *d*(Os-O3) = 1.994(4) Å (×2). BVS(Os) Were +3.37, +2.93, and +4.19, for which BVS = 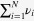, ν_*i*_ = exp[(*R*_0_−*d*_*i*_)/*B*], *N* was the coordination number, *B* = 0.37, *R*_0_(Os^5+^) = 1.868 [38].

The OsO_6_ framework of Bi_2.93_Os_3_O_11_ is tunnel-like, similar to K_0.84_OsO_3_, as shown in the inset of figure 2. The Bi atoms are not shown in the structural views for clarity. When assuming an ionic picture of the compounds, the formal Os valence should be +5.16 for K_0.84_OsO_3_ and +4.33 for Bi_2.93_Os_3_O_11_. This reflects the difference in the average Os–O bond distance of 1.964(8) Å and 1.992(1) Å for K_0.84_OsO_3_ and Bi_2.93_Os_3_O_11_, respectively. It is also reasonable to expect that the Os–Os distance in K_0.84_OsO_3_ is longer than that of Bi_2.93_Os_3_O_11_ owing to Coulomb repulsion between the positive charges; the observed Os–Os distance is indeed 3.7% longer than that of Bi_2.93_Os_3_O_11_. Nevertheless, the bond valence sum (BVS) was +4.63 for K_0.84_OsO_3_ and +4.29 for Bi_2._93Os3O11. Although the BVS(Os) for Bi_2.93_Os_3_O_11_ seems to be comparable to that of the ionic picture of +4.33, the BVS(Os) for K_0.84_OsO_3_ is poorly estimated. The poor estimation may be because K_0.84_OsO_3_ is not purely ionic. The Os–Os bonding could possibly be more significant in K_0.84_OsO_3_ than in Bi_2.93_Os_3_O_11_.

**Figure 3.**
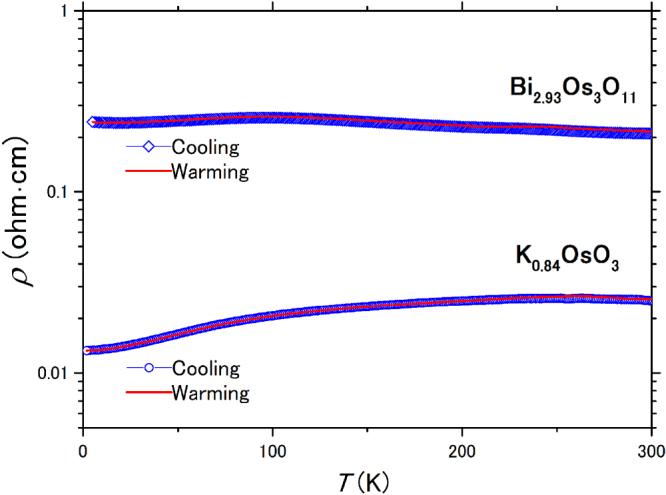
Temperature dependence of *ρ* of polycrystalline K_0.84_OsO_3_ and Bi_2.93_Os_3_O_11_.

The temperature dependence of *ρ* for a piece of polycrystalline K_0.84_OsO_3_ was measured (figure 3), and a metallic temperature dependence was found over the studied temperature range. Although *ρ* (∼0.025 ohm cm) at room temperature is approximately one order of magnitude higher than what is expected for a polycrystalline conducting oxide, the compound can still be characterized according to this type. In contrast, a piece of polycrystalline Bi_2.93_Os_3_O_11_ shows a weak temperature dependence and a *ρ* of ∼0.21 ohm cm at room temperature. However, this feature is not consistent with what is expected for a semiconducting oxide with an energy gap. Note that polycrystalline Bi_3_Os_3_O_11_ synthesized without a high pressure process shows a *ρ* of ∼0.001 ohm cm at room temperature [16], being remarkably lower than what was observed for the polycrystalline Bi_2.93_Os_3_O_11_. The true conductivity of Bi_2.93_Os_3_O_11_ is possibly masked by polycrystalline nature such as resistive grain boundaries and impurities. Additional studies on single crystals of the oxides are necessary to reveal true nature of electric transport. Attempts to grow crystals of the oxides under high-pressure and high temperature conditions have been unsuccessful so far.

**Figure 4.**
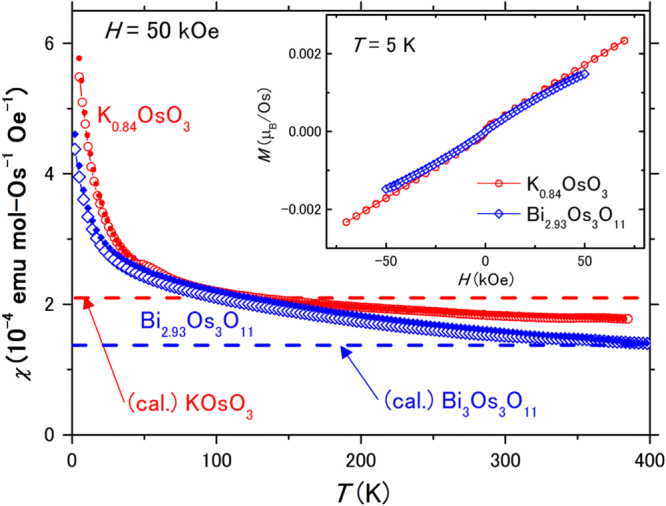
Temperature dependence of *χ* for polycrystalline K_0.84_OsO_3_ and Bi_2.93_Os_3_O_11_ in a magnetic field of 50 kOe. Solid and open symbols represent data measured in FC and ZFC conditions, respectively. The dashed lines indicate the theoretically calculated *χ* for stoichiometric hosts KOsO_3_ and Bi_3_Os_3_O_11_ for comparison. The inset shows the isothermal magnetizations of the compounds at 5 K.

The magnetic properties of polycrystalline oxides K_0.84_OsO_3_ and Bi_2.93_Os_3_O_11_ were studied and compared. Figure 4 shows the temperature dependence of *χ* for the oxides, revealing weakly temperature-dependent paramagnetic features over the temperature range. Any possible magnetic transition was unlikely over the measurements. Although a broad and small bump is seen at approximately ∼50 K for K_0.84_OsO_3_, any corresponding anomalies were not obvious in the *ρ* and *C*_p_ measurements (shown later). The inset shows the isothermal magnetizations of the oxides at low temperature (5 K) and only quasi-linear behaviors with trivial magnetizations were observed. Therefore, we tentatively assumed that the magnetic bump is likely impurity driven. The overall magnetic measurements suggested that the compounds are both paramagnetic at temperatures above 2 K. The temperature dependence of *χ* at the low temperature limit remains unconnected to any magnetic model and impurities.

**Figure 5.**
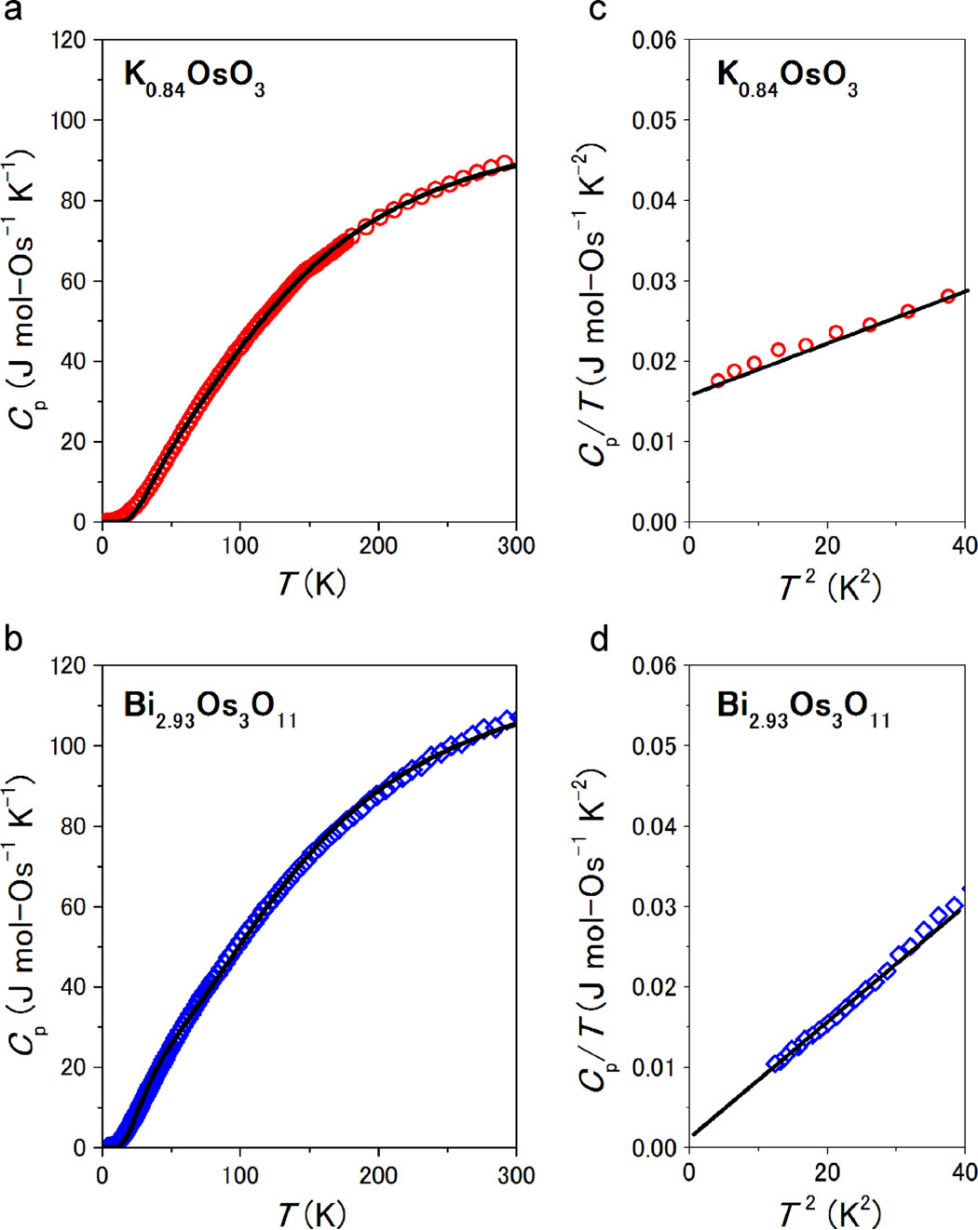
Temperature dependence of *C*_p_ of polycrystalline (a) K_0.84_OsO_3_ and (b) Bi_2.93_Os_3_O_11_. The solid line represents the fitting curve. A linear fit of the low-temperature measurements of the *C*_p_*/T* versus *T*^2^ plot is shown for (c) K_0.84_OsO_3_ and (d) Bi_2.93_Os_3_O_11_, respectively.

Specific heat measurements for the oxides were conducted; their *C*_p_ versus *T* curves are shown in figures 5(a) and (b). Over the temperature range, *C*_p_ varies monotonically and any indicative anomaly for a transition is unobvious. The *C*_p_ versus *T* curve was analyzed by a linear combination of the Debye and Einstein model, as was conducted for related materials [40]. The analytical formula was

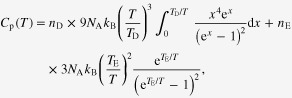
where *N*_A_ is Avogadro’s constant, and *T*_D_ and *T*_E_ are the Debye and Einstein temperatures, respectively. The scale factors, *n*_D_ and *n*_E_, correspond to the number of vibrational modes per formula unit in the Debye and Einstein models, respectively. The fitting curves yielded the following: *T*_D_ of 628(7) K, *T*_E_ of 150(2) K, *n*_D_ of 2.88(3), and *n*_E_ of 1.25(3) for K_0.84_OsO_3_; and *T*_D_ of 650(7) K, *T*_E_ of 110(2) K, *n*_D_ of 3.63(2), and *n*_E_ of 1.34(3) for Bi_2.93_Os_3_O_11_. The Einstein term added to the Debye term increased the quality of fitting, suggesting a possibility that the phonon density of state (DOS) forms a rather complex structure over the whole temperature range. However, analysis on the *C*_p_/*T* ^3^ versus *T* plots (not shown) indicated that anomalous Einstein contributions, which are indicative of lattice rattling, were not obvious (unlike related oxides) [40]. Further analysis of the phonon modes is needed to clarify the phonon DOS structure of both the compounds.

The low-temperature measurements of the *C*_p_/*T* versus *T*^2^ plot for each compound were analyzed by an approximated Debye model (figures 5(c) and (d)), which was *C*_p_/*T* = *βT*^2^ + *γ*, where *β* and *γ* are a constant and the Sommerfeld coefficient, respectively. The fit of the plots yielded *β* = 3.03(9) × 10^−4^ J (one mole of osmium atoms (mol-Os))^−1^ K^−4^ and *γ* = 16.8(2) mJ mol-Os^−1^ K^−2^ for K_0.84_OsO_3_; and *β* = 7.00(9) × 10^−4^ J mol-Os^−1^ K^−4^ and *γ* = 1.6(2) mJ mol-Os^−1^ K^−2^ for Bi_2.93_Os_3_O_11_. The *T*_D_ for K_0.84_OsO_3_ and Bi_2.93_Os_3_O_11_ were calculated from *β* of 315(4) K and 250(1) K, respectively. It appeared that the *γ* for Bi_2.93_Os_3_O_11_ is nearly one tenth of the *γ* for K_0.84_OsO_3_; we therefore carefully investigated the electronic state of the compounds by a theoretical method.

**Figure 6.**
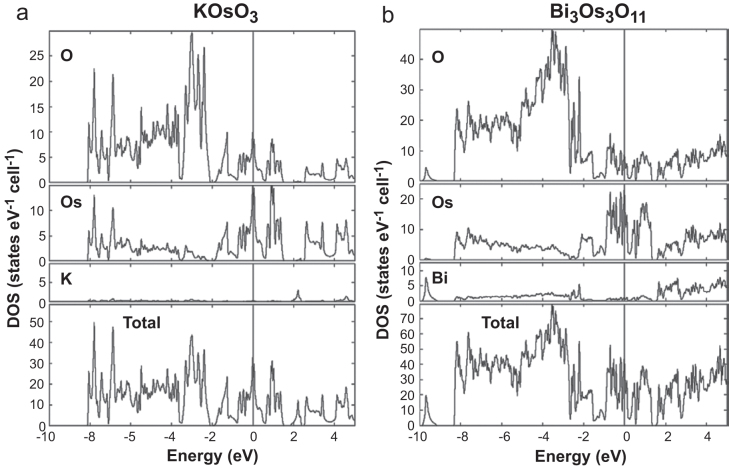
Electronic DOS of stoichiometric hosts of (a) K_0.84_OsO_3_ and (b) Bi_2.93_Os_3_O_11_. The vertical line indicates *E*_F_.

Figures 6(a) and (b) show the theoretically predicted electronic DOS structure for the stoichiometric hosts K_0.84_OsO_3_ and Bi_2.93_Os_3_O_11_, respectively. The total DOS was found to consist of mainly Os and O contributions and little from K/Bi. Both compounds have a nontrivial electronic DOS at the Fermi level (*E*_F_). Therefore, the hosts are expected to be metallic. The estimated *γ* for KOsO_3_ from the DOS at *E*_F_ is 15.6 mJ mol-Os^−1^ K^−2^, which is nearly comparable to the observed *γ* for K_0.84_OsO_3_ (16.8(2) mJ mol-Os^−1^ K^−2^). However, the estimated *γ* for Bi_2.93_Os_3_O_11_ is 9.7 mJ mol-Os^−1^ K^−2^, which is much larger than the observed *γ* for Bi_2.93_Os_3_O_11_ (1.6(2) mJ mol-Os^−1^ K^−2^). This disagreement between the expected and observed *γ* for the compounds is possibly owing to a steep change of DOS in the vicinity of *E*_F_ with lowering the Bi stoichiometry. This phenomenon has been previously discussed for related Os oxides [41]. Although we need to carefully investigate possible contributions from spin-polarization and spin–orbit interactions over the DOS structure, opening a full gap at *E*_F_ for Bi_2.93_Os_3_O_11_ seems unlikely.

## 4. Conclusions

Materials with 5*d* electrons may show characteristic features owing to radially extended valence orbitals and large spin–orbit couplings of 5*d* electrons over 3*d* electrons. For example, a perovskite-type oxide, NaOsO_3_, shows a Slater-like transition [30, 31] and a LiNbO_3_-type oxide, LiOsO_3_, shows a ferroelectric-like transition in the metallic state [29]. The results lead to the reasonable expectation that KOsO_3_, if synthesized, also shows characteristic features of 5*d* electrons. Nevertheless, KSbO_3_-type crystalline K_0.84_OsO_3_ shows only a weak temperature-dependent paramagnetic feature. In addition, KSbO_3_-type Bi_2.93_Os_3_O_11_ was also synthesized under high-pressure and high-temperature conditions. The Os–O network in this system was found to form a similar tunnel motif to that of K_0.84_OsO_3_. Although the formal valence of Os decreased from +5.16 (K) to +4.40 (Bi), the observed magnetic and electronic properties did not change significantly.

In contrast to the remarkable 5*d* properties of related compounds NaOsO_3_ [30, 31] and LiOsO_3_ [29], K_0.84_OsO_3_ and Bi_2.93_Os_3_O_11_ seem to have less characteristic 5*d* properties above 2 K. Both compounds showed rather weak temperature-dependent paramagnetism and metallic transports. Disorders such as K/Bi vacancies and the polycrystalline nature of the compounds (including grain boundaries and impurities) could possibly complicate the observed 5*d* properties. Nevertheless, the magnetic and electronic properties are quite similar to what was observed for structurally relevant 5*d* oxides, including Bi_3_Re_3_O_11_ (Re^4.33+^; 5*d* ^2.66^) [18] and Ba_2_Ir_3_O_9_ (Ir^4.66+^; 5*d* ^4.33^) [26], regardless of the number of 5*d* electrons. The absence of a long-range magnetic order seems to be common among the tunnel-like structures of 5*d* oxides. Further studies on high-quality single crystals of newly synthesized KSbO_3_-type material K_0.84_OsO_3_ may reveal the 5*d* characteristic features and help establish a comprehensive picture of the 5*d* electronic system.
